# Objective Smartphone Screen Time Monitoring Among Caregivers of Preschool-Aged Children: Reliability Study of Monitoring Duration

**DOI:** 10.2196/82846

**Published:** 2026-09-10

**Authors:** Joshua Culverhouse, Taylor Adair, Meghan Restino, Heidi M Weeks, Jenny S Radesky, R Glenn Weaver, Sarah Burkart, Hannah Parker, Olivia L Finnegan, Anthony J Holmes, Keagan Kiely, Rahul Ghosal, Srihari Nelakuditi, Michael W Beets, Bridget Armstrong

**Affiliations:** 1 Department of Exercise Science, University of South Carolina, PHRC - Room 140, 921 Assembly St, Columbia, SC, 29201, United States, 1 8032017927; 2 University of Michigan School of Public Health, Ann Arbor, MI, United States; 3 University of Michigan Medical School, Ann Arbor, MI, United States; 4 Department of Epidemiology and Biostatistics, University of South Carolina, Columbia, SC, United States; 5 Department of Computer Science and Engineering, University of South Carolina, Columbia, SC, United States

**Keywords:** screen time, passive sensing, smartphone, sampling duration, reliability

## Abstract

**Background:**

Reliable measurement of smartphone screen time is important for research examining associations between digital media use and health outcomes. Although objective monitoring tools reduce bias associated with self-report, the number of monitoring days required to reliably estimate typical smartphone use remains unclear. This question is particularly important for study design because longer monitoring periods increase participant burden and the likelihood of missing data.

**Objective:**

This study aimed to determine the minimum number of valid monitoring days within a 7-day measurement period required to reliably estimate average daily smartphone screen time during the same 7-day period among primary caregivers of preschool-aged children using 2 objective methods.

**Methods:**

This secondary analysis included caregivers participating in 2 observational studies conducted in the Eastern United States. Android users installed Chronicle, a passive sensing application that records raw Android UsageEvents data, which were preprocessed into smartphone session events (pickups where the device was unlocked). iOS users submitted daily screenshots of Apple Screen Time summaries. Reliability analyses included 100 Android Chronicle participants and 129 iOS Screenshot participants with at least 7 consecutive complete days of data. The primary outcome was day-level total smartphone screen time. For Android Chronicle, this was defined as the summed duration of device-unlock sessions across each calendar day, whereas for iOS Screenshot, this was defined as the daily total reported by Apple Screen Time. One-way random-effects intraclass correlation coefficients (ICC [1,1]) with 95% bootstrap CIs (500 resamples) were calculated separately for Android and iOS data. The Spearman-Brown prophecy formula was used to estimate the number of monitoring days required to achieve target reliability thresholds (ICC≥0.70, 0.80, and 0.90). Sensitivity analyses using the combined durations of sessions and glances produced similar conservative monitoring-day recommendations.

**Results:**

Single-day ICCs were 0.69 (95% CI 0.63-0.76) for Android Chronicle and 0.71 (95% CI 0.65-0.77) for iOS Screenshot data. Point-estimate projections indicated that 2 monitoring days were sufficient to achieve an ICC≥0.80 for both data sources. Using the more conservative criterion that the lower bound of the projected 95% CI exceeded 0.80, 3 monitoring days were required for both Android and iOS data. Screen time was significantly lower on weekends than on weekdays, suggesting that monitoring periods should include at least 1 weekend day.

**Conclusions:**

Among caregivers of preschool-aged children, relatively short objective monitoring periods may provide reliable estimates of average daily smartphone screen time within a 7-day monitoring period. Two monitoring days were sufficient under point-estimate projections, whereas 3 days were required using a conservative lower-bound criterion. These findings support the feasibility of brief smartphone monitoring protocols for estimating the 7-day average duration, while emphasizing the importance of including weekend representation and avoiding the extension of these estimates to longer-term habitual smartphone behavior or screen time metrics beyond duration without further evidence.

## Introduction

### Background

Smartphone ownership is nearly universal in the United States, with more than 90% of adults owning a smartphone [1]. Alongside rising usage, growing evidence links smartphone use to a range of health and psychosocial outcomes [2-4]. However, progress in this area depends on the quality of the measures used to quantify smartphone behavior. In digital media research, screen time and related exposure variables are commonly used as predictors or outcomes, but many studies have relied on retrospective self-report measures [5-7].

### Limitations of Self-Reported Smartphone Use

Self-reported media use is vulnerable to recall error, social desirability bias, and difficulty estimating frequent behaviors embedded in daily routines [8,9]. These limitations may be especially relevant for smartphone use, which often occurs through short, repeated interactions across varied contexts and alongside other activities [10]. A systematic review and meta-analysis found that self-reported digital media use correlated only moderately with logged use and was rarely an accurate reflection of objectively recorded behavior [7]. This suggests that self-reported and logged measures may not be interchangeable and supports the use of objective measurement when estimating smartphone exposure.

### Objective Smartphone Measurement

Objective smartphone measures generally fall into several related approaches. On Android devices, timestamped operating-system event logs can be processed to construct smartphone use behaviors such as sessions, glances, and app use episodes [11]. Research tools such as Chronicle and the Effortless Assessment Research System (EARS) use these types of passive sensing data to provide detailed measures of device or app interaction [12-14]. Other intensive approaches, such as Screenomics, capture repeated screenshots to characterize digital context and content, offering rich behavioral detail but with greater privacy, storage, and processing demands [15]. In contrast, iOS research often relies on less granular approaches or participant-submitted screenshots of Apple Screen Time summaries because Apple restricts third-party access to detailed device activity data. Although these objective approaches reduce reliance on retrospective self-report, they still require decisions about the number of monitoring days needed to obtain reliable estimates.

### Within-Person Variability and Monitoring Duration

Smartphone use also varies meaningfully within individuals across days and situations. Recent work distinguishing person-specific from situation-specific variation in media use found that a large share of media use variance occurs within persons across situations, underscoring the need for repeated measurement designs rather than single time point estimates [16]. At the same time, smartphone behavior may include habitual or automatic interactions triggered by contextual cues, making it difficult for participants to accurately recall or summarize their use [17]. Together, these features create a practical measurement challenge: researchers need objective monitoring protocols that are long enough to capture typical use, but not so long that they increase participant burden or missing data.

Prior studies have addressed similar questions using correlation-based approaches, examining the association between short-term and longer-term monitoring windows [18,19]. For instance, Wilcockson et al [18] found that 5 days of monitoring were sufficient to characterize weekly smartphone duration and 2 days were sufficient for checking behavior (pickups lasting <15 s). Although informative, correlations primarily capture rank-order consistency across individuals and do not directly estimate measurement reliability, absolute agreement, or the contribution of within-person variability.

### Reliability-Based Study Planning

An alternative approach uses intraclass correlation coefficients (ICCs) and the Spearman-Brown prophecy formula [20,21]. ICC-based approaches are useful for planning measurement protocols because they quantify the proportion of observed variance attributable to stable between-person differences, provide an estimate of measurement error in the exposure, and can be paired with the Spearman-Brown formula to estimate how many repeated days are needed to achieve a prespecified reliability threshold. These methods are widely used in physical activity and other behavioral monitoring research to determine the minimum monitoring periods required for stable estimates of behavior [22-26], but have not been widely applied to objective smartphone screen time measurement.

### Study Aim

In this study, we used the ICC and Spearman-Brown methods to estimate the minimum number of valid monitoring days required, within a 7-day monitoring period, to reliably estimate average daily smartphone screen time duration among primary caregivers of young children. Separate analyses were conducted for 2 objectively collected data sources: (1) passive sensing via the Chronicle app on Android devices and (2) screenshots of Apple Screen Time on iOS devices. Because these data sources reflect different platform-specific measurement approaches and were collected from distinct participant groups, the aim was not to test equivalence or directly compare Android and iOS estimates. Rather, our aim was to provide data-source–specific reliability estimates and practical guidance for researchers studying smartphone behavior in this population, where some days of monitoring may be missing, invalid, or incomplete.

## Methods

### Participants and Study Design

This secondary analysis used data from 2 observational studies involving caregivers of preschool-aged children: a pilot feasibility study [27] and an ongoing longitudinal cohort study [28] (R01HD112311). Both studies recruited nonrandom samples via social media across the eastern United States and used identical smartphone screen time assessment protocols. Inclusion criteria were (1) primary caregiver of a child aged 3 to 5 years, (2) ownership of a smartphone, and (3) ability to read and speak English. Remote data collection included surveys, ecological momentary assessments, and passive smartphone sensing.

Pilot study data were collected from September 2020 to February 2022 using a 30-day monitoring protocol, whereas cohort study data were collected from September 2024 to May 2025 using a 14-day monitoring protocol. Android Chronicle data were available from both studies, whereas iOS Screenshot data were collected only in the cohort study.

### Ethical Considerations

Both parent studies were approved by the University of South Carolina Institutional Review Board (IRB) in August 2020 (Pro00092634) and December 2023 (Pro00133829). All participants provided informed consent before participation, and the IRB approvals covered secondary analyses of the collected data. Participant data were deidentified before analysis and stored on secure, access-restricted university servers. Smartphone data were processed using participant identifiers rather than names or directly identifying information. Participants received up to US $440 per assessment period for completing the broader study protocol, including parent surveys, daily diaries, ecological momentary assessments, and other remote data collection procedures. Smartphone screenshot submissions were completed as part of the compensated daily diary protocol (US $5 per completion). Chronicle data collection was not directly compensated.

### Smartphone Screen Time Measurement

#### Android Chronicle

Android users installed Chronicle, a passive sensing application that records timestamped device interaction events [12]. Chronicle accesses raw Android event-log data through the Android UsageEvents framework, which captures application transitions, screen interactions, and device state events [13]. Data are uploaded at regular intervals to a secure server when the device is connected to Wi-Fi.

**Figure 1.**
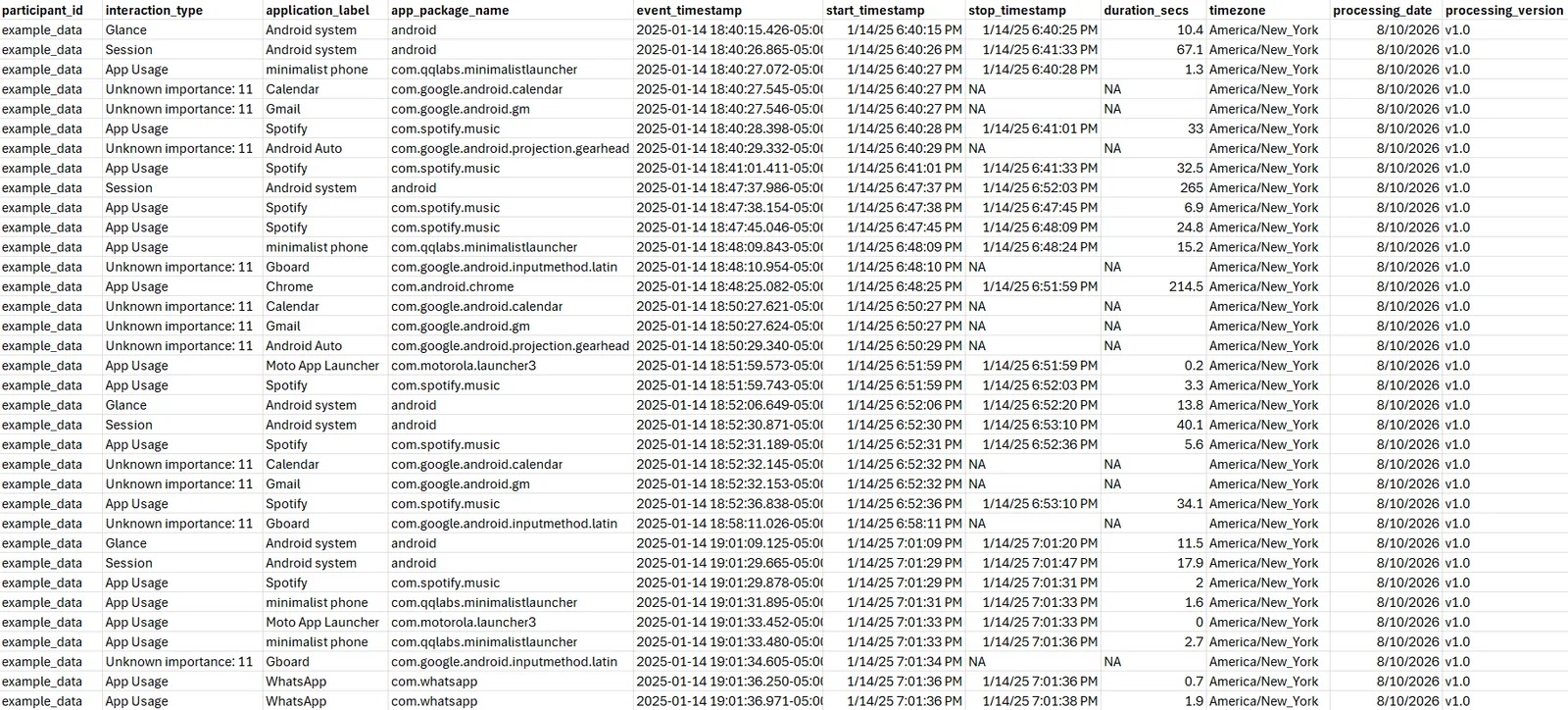
Example preprocessed Chronicle output.

For the current analysis, raw Android event logs were used to derive smartphone interaction measures based on an adapted implementation of the framework proposed by Parry and Torth [11] for constructing meaningful smartphone use behaviors from Android event streams. This approach constructed smartphone sessions (device-unlock episodes), glances (brief screen activations without unlocking), and foreground app use episodes from timestamped event sequences (Figure 1).

The primary Android outcome was total daily smartphone session duration, aggregated by each calendar day. In a sensitivity analysis, we also examined combined session and glance duration. Conservative monitoring-day recommendations were unchanged; therefore, the primary analyses presented focus on session-derived estimates.

#### iOS Screenshot

**Figure 2.**
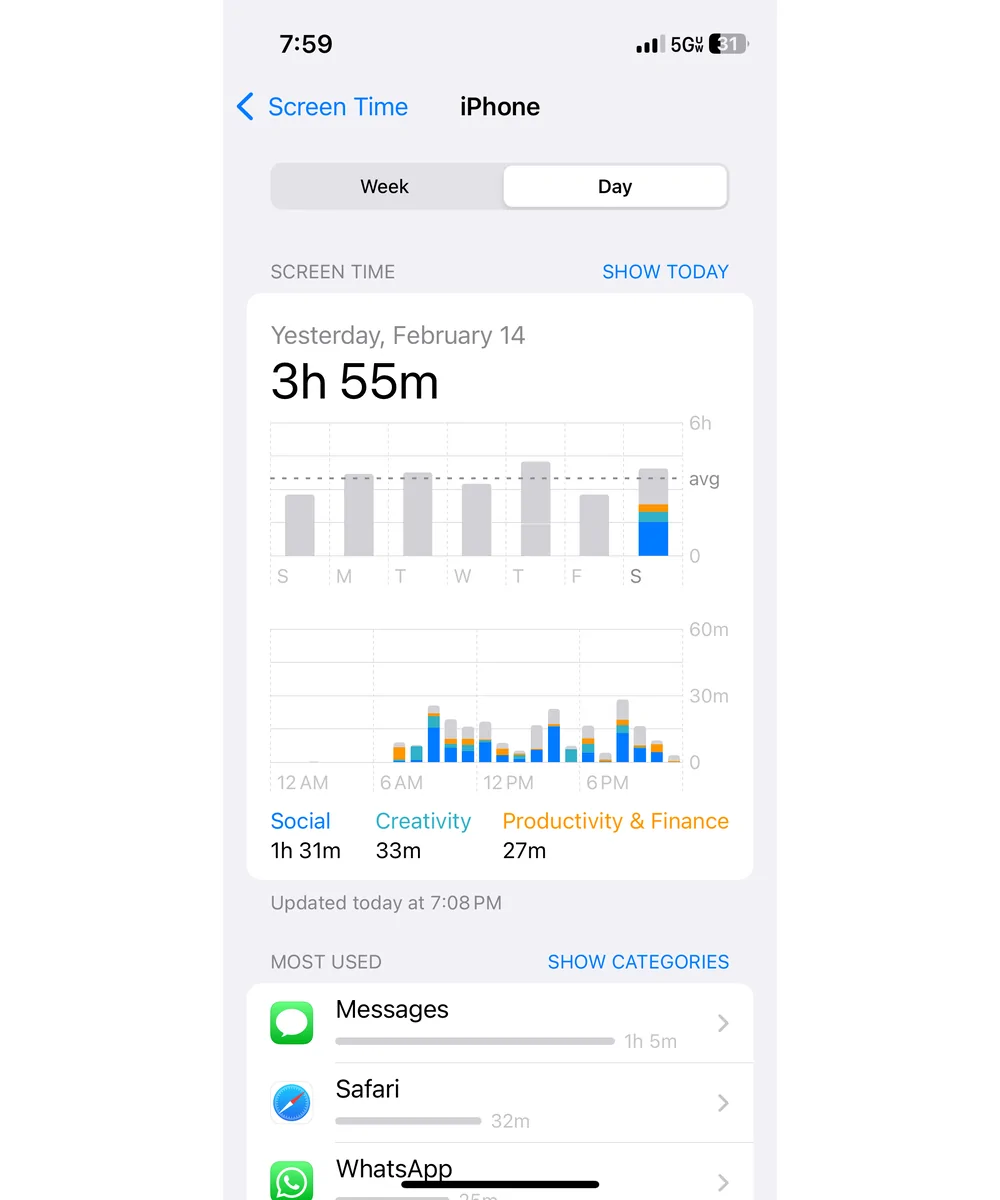
Example iOS Screenshot (Apple Screen Time).

Comparable passive sensing data were not available on iOS devices because Apple restricts third-party access to detailed app activity data. Therefore, iOS users submitted daily screenshots of their previous day’s built-in Apple Screen Time summary via a daily diary survey link texted to their smartphones (Figure 2). Surveys were administered through Qualtrics and automatically distributed using an internal messaging system (WearData).

Screenshots were manually reviewed for correct date, device, and completeness. They were excluded if the displayed date did not match the target reporting day (submitted day minus 1), if the image was a duplicate submission for an already processed day, if the image was unclear or unreadable, or if the hourly bar chart was incomplete or could not be reliably extracted. When multiple valid screenshots were submitted for the same participant-day, only one screenshot was retained. These checks were completed before aggregation to daily totals. We then applied a custom open-source Python script [29] to extract per-hour screen time values from the screenshot bar chart. Hourly values were summed to generate total daily screen time. To validate this approach, we compared the extracted totals against the daily totals displayed on a random sample of 100 screenshots. The mean difference was −2.7±11.7 minutes per day (relative difference of −0.01%±0.04%), with a Lin concordance coefficient of 0.998, demonstrating excellent agreement. This validation confirmed the accuracy of extracting and summing hourly values from submitted screenshots relative to the daily total displayed by Apple Screen Time. However, it should not be interpreted as criterion validation of Apple Screen Time against raw device logs because Apple’s underlying screen time definitions and algorithms are proprietary.

### Data Processing and Cleaning

Android Chronicle data were processed using a reproducible preprocessing and cleaning pipeline. Raw Android event logs were first transformed into structured smartphone interaction events, including sessions, glances, and foreground app use episodes [30]. Although this study used session-derived duration measures as the primary outcome, app use episodes were retained during preprocessing and cleaning to support transparent reconstruction of smartphone interaction events and future analyses. Cleaning procedures then standardized timestamps and time zones, collapsed adjacent split app use segments, truncated predefined low-engagement or background applications, identified implausibly long events, and flagged incomplete or potentially unreliable days [31].

To reduce inflation of duration estimates, predefined low-engagement applications (eg, launcher, clock, screensaver, or other background-system applications) were truncated to a maximum duration of 10 minutes rather than excluded entirely. The truncation threshold was chosen in line with prior work using Chronicle data [13]. A list of these apps is available with the cleaning script linked above. Events with implausibly long durations (>3 h) were reviewed using prespecified rules based on Google Play Store app categories (GenreID), with prolonged durations considered plausible for “video players,” “entertainment,” “social,” and “games” apps. All events lasting for more than 6 hours were considered implausible and truncated to 10 minutes. Prolonged inactivity gaps (>12 h without recorded Android events or device shutdown markers) were treated as likely periods of missing data. Days containing such gaps, as well as partial start and end days, were excluded from analysis.

To summarize the application of these rules, we examined the exclusion of partial or incomplete days across all available Chronicle records processed before analytic window selection. Across these records, 10% of participant-days were excluded because of partial coverage or prolonged inactivity gaps. To summarize the impact of long-session truncation on the data used in the primary reliability analyses, we examined the final 7-day analytic windows. Within these analytic windows, 9 of 64,644 pickup/session events (0.01%) exceeded 6 hours and were truncated. These rules were applied systematically using prespecified criteria to reduce implausible inflation of screen time estimates. We did not interpret uncleaned values as valid alternative estimates because they included incomplete days and anomalously long events that were considered likely data artifacts.

Screenshot-based iOS data required only verification of complete 24-hour coverage and of valid screenshot formatting before aggregation into daily totals. For the current analysis, we included only participants with at least 7 consecutive complete days of objective smartphone use data after cleaning. For participants with more than 7 consecutive complete days available, we selected the first 7 days from the first qualifying consecutive sequence after cleaning. This rule was applied consistently across participants and data sources to define a reproducible 7-day analytic window.

### Statistical Analysis

#### Overview

We fitted a linear mixed-effects model (lme4) with data source (iOS vs Android), day type (weekend vs weekday), and their interaction as fixed effects, and a random intercept for participant to account for repeated daily observations within individuals. Fixed-effect estimates (*B*), standard errors (SEs), 95% CIs, and *P* values are reported.

We conducted separate analyses for Android Chronicle and iOS Screenshot data because these data sources were derived from different platform-specific measurement approaches. The aim was not to directly compare Android and iOS screen time estimates but to provide data-source–specific reliability estimates for 2 objective approaches to measuring daily smartphone use duration.

The primary outcome for both data sources was day-level total smartphone screen time in hours. In the Android Chronicle data, this was defined as the summed duration of device-unlock sessions for each calendar day. In the iOS Screenshot data, this was defined as the daily total reported by Apple Screen Time and extracted from participant-submitted screenshots. The following approaches were used, as described in the following sections.

#### ICC

A 1-way random-effects ICC for single measures with absolute agreement (ICC [1,1]) was calculated to quantify the proportion of variance attributable to stable between-person differences in daily screen time duration [20]. ICC (1,1) was selected because the aim was to estimate the reliability of a single day of monitoring sampled from a broader universe of possible days, rather than agreement between a fixed set of raters or measurement occasions. This provides a direct estimate of test-retest reliability for a single day of monitoring. ICCs were computed using the *irr::icc*() function in R (R Foundation for Statistical Computing) [32]. To quantify uncertainty around the single-day ICC estimate, we generated a 95% CI using nonparametric bootstrapping (resampling participants with replacement; 500 replicates). For each bootstrap replicate, the single-day ICC estimate was entered into the Spearman-Brown prophecy formula to generate projected ICC*_k_* values and corresponding estimates of the number of monitoring days required to achieve target reliability thresholds, thereby propagating uncertainty from the single-day ICC through all projected reliability estimates.

#### Spearman-Brown Prophecy Formula

To estimate how reliability improves with additional days, we calculated the projected expected ICC for *k*=2 to 7 days using the following formula:


$${ICC}_{k}=\;\frac{k\cdot {ICC}_{1}}{1+\left(k-1\right)\cdot {ICC}_{1}}$$

where ICC_1_ is the reliability from a single day of monitoring, and ICC_*k*_ is the projected reliability for *k* days [21,33]. Then, for each bootstrap draw of ICC_1_*,* we applied the following formula to estimate the number of monitoring days needed to reach thresholds of ICC=0.70, 0.80, and 0.90:


$$k^{*}=\;\frac{ICC_{target}\left(1-{ICC}_{1}\right)\;}{{ICC}_{1}\left(1-ICC_{target}\right)}$$

Here, ICC_target_ is the desired reliability level (0.70, 0.80, or 0.90), and k^*^ is the projected number of days needed to achieve that level. We summarized the resulting k^*^ distribution as the median (95% CI) and showed the full ICC vs days curves (with 95% bootstrap CI). For interpretation, we focus on the point at which the lower bound of the 95% CI of the projected ICC curve first reaches ≥0.80, a commonly accepted threshold for adequate reliability in behavioral research [34]. Projected monitoring durations were rounded up to the nearest whole day for practical interpretation.

The Spearman-Brown projections assume that repeated monitoring days function as exchangeable indicators of the same underlying average daily behavior. This assumption may be imperfect for smartphone screen time because use can vary systematically by day type. Therefore, the projected monitoring durations were interpreted as estimates of the number of valid days needed to estimate the average daily smartphone duration within the observed 7-day monitoring period. They were not interpreted as evidence that the same number of days would reliably estimate longer-term habitual smartphone use across weeks, seasons, or other contexts.

To assess potential differences between included and excluded participants, we compared available demographic characteristics by inclusion status within each data source. Because only 7 Android Chronicle participants were excluded, Android comparisons were interpreted descriptively. For iOS Screenshot participants, age was compared using a 2-tailed Welch *t* test, and categorical characteristics were compared using Fisher exact test. All statistical analyses were conducted in R (version 4.4.0), and statistical significance was set at *P*<.05.

### Reporting Guidelines

This manuscript was reviewed using the STROBE (Strengthening the Reporting of Observational Studies in Epidemiology) checklist for observational studies. The completed checklist is provided in Checklist 1.

## Results

### Participant Characteristics

Of the 316 participants with objective smartphone use data available for screening, 229 (72.5%) were included in the final analytic sample (Table 1). For Android (Chronicle), 100 of 107 participants (93.5%) had at least 7 consecutive valid days after cleaning. For iOS Screenshot, 129 of 209 participants (61.7%) had at least 7 consecutive valid screenshots. Because only 7 Android Chronicle participants were excluded, included vs excluded comparisons were interpreted descriptively. Among iOS Screenshot participants, included and excluded participants did not differ significantly by age, sex, ethnicity, employment, or income (all *P*>.05), but differed by race (*P*=.001) and education (*P*=.002). Included iOS participants were more likely to be White and to have a college degree or higher than excluded iOS participants.

**Table 1.** Participant inclusion by smartphone data source.

Data source	Available for screening, n	Included, n (%)	Excluded, n (%)
Android Chronicle	107	100 (93.5)	7 (6.5)
iOS Screenshot	209	129 (61.7)	80 (38.3)
Total	316	229 (72.5)	87 (27.5)

For the final analytic sample, the mean age was 36.4 years (SD 5.7), and the sample was predominantly female primary caregivers (218/229, 95.2%), which is typical of child and family-based research [35]. Because data sources were platform-specific, Android and iOS users represent distinct subsamples, with some demographic differences (Table 2). In particular, iOS Screenshot participants were more likely to have a college degree or higher, be employed, and report household income of US $100,000 or more. These differences should be considered when interpreting any descriptive comparisons between platform and data-source groups. However, these differences were not adjusted for, as the focus of this analysis was on within-sample measurement reliability rather than between-group comparisons.

**Table 2.** Participant demographics by smartphone screen time data source.

Characteristics	Android Chronicle (n=100)	iOS Screenshot (n=129)
Age (y), mean (SD)	36.6 (6.8)	36.3 (4.7)
Sex, n (%)		
Female	94 (94)	124 (96.1)
Race, n (%)		
Black	19 (19)	15 (11.6)
White	74 (74)	104 (80.6)
Asian, more than 1 race, or other	7 (7)	10 (7.8)
Hispanic ethnicity (yes), n (%)	5 (5)	8 (6.2)
Education^a^, n (%)		
High school or less	11 (11)	3 (2.3)
Some college or 2-year degree	39 (39)	20 (15.5)
College graduate or higher	50 (50)	106 (82.2)
Employment^a^, n (%)		
Employed	54 (54)	98 (76.0)
Student	28 (28)	19 (14.7)
Unemployed	11 (11)	5 (3.9)
Other	7 (7)	7 (5.4)
Income^a^, n (%)		
<US $20,000	10 (10)	2 (1.6)
US $20,000-$49,000	23 (23)	13 (10.1)
US $50,000-$99,000	39 (39)	39 (30.2)
≥US $100,000	28 (28)	75 (58.1)

a Android Chronicle and iOS Screenshot participants differed on some demographic characteristics. In particular, iOS Screenshot participants were more likely to have a college degree or higher, be employed, and report household income ≥US $100,000.

### Data Source and Day Type Differences

A linear mixed-effects model with a random intercept for participant, including both data source and day type as fixed effects, showed no significant difference in mean daily screen time between iOS and Android users (*B*=15.7 min, SE=21.4, 95% CI –26.2 to 57.5; *P*=.46). However, screen time was significantly lower on weekends than on weekdays (*B*=–16.1 min, SE=5.6, 95% CI –27.0 to –5.2; *P*=.004). The interaction between data source and day type was not significant (*B*=6.5 min, SE=11.3; *P*=.56), indicating that the weekend-weekday difference did not vary by data source. Reliability analyses were conducted across all 7 monitoring days rather than separately by day type because the aim was to estimate the reliability of a typical week of smartphone use. Mean daily screen time by day of the week is shown in Multimedia Appendix 1.

### Reliability and Monitoring Duration

**Figure 3.**
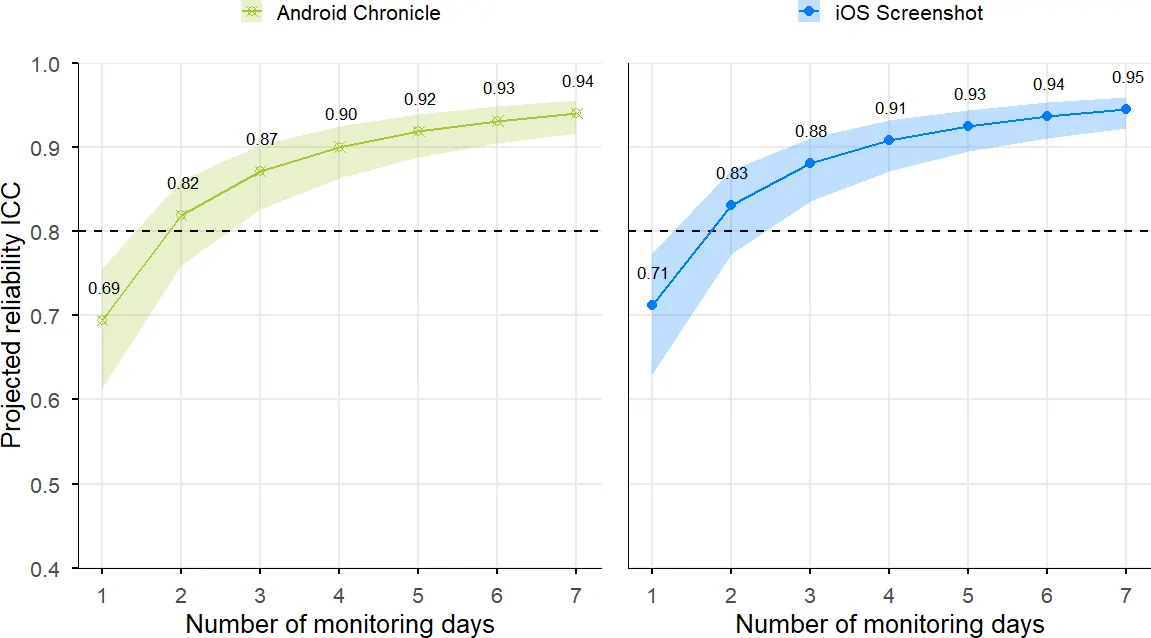
Projected reliability of average daily smartphone screen time by number of monitoring days, estimated using the Spearman-Brown prophecy formula. Points show projected intraclass correlation coefficients (ICC [1,1]) estimates for 1 to 7 monitoring days, shaded bands show 95% CIs, and the dashed horizontal line indicates ICC=0.80.

Table 3 presents the reliability results for daily smartphone screen time. For Android Chronicle data, the single-day ICC was 0.69 (95% CI 0.63-0.76). For iOS Screenshot data, it was 0.71 (95% CI 0.65-0.77). For interpretability, projected monitoring durations were rounded up to the nearest whole day when deriving practical recommendations. Using Spearman-Brown projections based on the point estimates, 2 monitoring days were sufficient for both Android Chronicle and iOS Screenshot data to achieve projected ICC≥0.80 (Figure 3). Using the more conservative lower-bound approach, 3 days were required for both data sources. Sensitivity analyses using combined session and glance duration produced a single-day ICC of 0.64 (95% CI 0.57-0.72). The projected monitoring durations were 1.3 (95% CI 1.0-1.7) days, 2.2 (95% CI 1.7-2.9) days, and 5.0 (95% CI 3.9-6.6) days for ICC thresholds of 0.70, 0.80, and 0.90, respectively. After rounding, both the point-estimate and conservative recommendations were 3 days to achieve ICC≥0.80.

**Table 3.** Single-day intraclass correlation coefficients (ICCs; 95% bootstrapped CI), and projected number of days needed for target reliabilities of 0.7, 0.8, and 0.9, by data source.

Data source	Single-day ICC (95% CI)	Projected number of days (95% CI)			Between-person variance	Within-person variance	Practical recommendation for ICC≥0.80^a^
		ICC=0.7	ICC=0.8	ICC=0.9			
Android Chronicle (n=100)	0.69 (0.63-0.76)	1.0 (0.8-1.3)	1.8 (1.4-2.3)	4.0 (3.1-5.1)	26,788	11,804	Point estimate: 2 days; conservative: 3 days
iOS Screenshot (n=129)	0.71 (0.65-0.77)	0.9 (0.7-1.3)	1.6 (1.2-2.2)	3.6 (2.8-4.8)	22,265	9018	Point estimate: 2 days; conservative: 3 days

a Projected monitoring durations were rounded up to the nearest whole day. Conservative recommendations were based on the lower bound of the 95% CI for projected ICC estimates.

## Discussion

### Principal Findings

This study examined the minimum number of monitoring days required to reliably estimate average daily smartphone screen time among primary caregivers of preschool-aged children using objective Android and iOS monitoring approaches. Daily screen time duration estimates demonstrated high reliability even with a small number of days. When using the point estimate, projections indicated that 2 monitoring days were sufficient for both Android Chronicle and iOS Screenshot data to achieve ICC≥0.80. When using the more conservative lower-bound criterion of the 95% CIs, 3 days were required for both data sources. These estimates should be interpreted as the number of valid days needed to estimate average daily smartphone duration within a 7-day monitoring period. Although the weekday-weekend difference was modest in absolute terms, screen time differed systematically by day type. Therefore, shortened protocols should include weekend representation. For a 3-day protocol, this would most pragmatically involve at least 2 weekdays and 1 weekend day.

Our findings align with prior research in other forms of objective behavioral monitoring, such as physical activity, where 3 to 7 days of accelerometer wear are typically recommended to obtain reliable estimates of movement behaviors [22-26]. Interestingly, smartphone screen time appears to require even fewer days, potentially reflecting a degree of stability of daily phone use, at least among primary caregivers.

Previous studies that estimated the number of days needed for reliable smartphone measurement relied on correlation-based analyses [18,19]. For instance, Wilcockson et al [18] found that 5 days were sufficient to characterize weekly usage using Pearson correlations. Although these studies provide valuable early insights, correlation-based methods primarily reflect rank-order consistency and do not directly quantify measurement reliability. In other words, correlations measure whether individuals maintain the same relative position over time, but do not quantify measurement reliability in a statistical sense. In contrast, our approach uses ICCs, which directly estimate the proportion of total variance attributable to stable between-person differences in average daily screen time over a week [20]. Combined with the Spearman-Brown formula, this approach allows us to estimate how many days of monitoring are needed to achieve a desired level of reliability (eg, ICC≥0.80) while accounting for both within-person and between-person variability in screen time [34]. Although our results are similar to earlier findings, they provide a methodologically rigorous basis for these recommendations by directly estimating measurement reliability. This strengthens confidence in the use of short monitoring periods for future study design.

Objective methods for monitoring smartphone use have progressed rapidly, with built-in summaries (eg, iOS Screenshot) and passive sensing tools (eg, Android Chronicle) each presenting distinct advantages and trade-offs [36]. Built-in summaries, such as Apple’s Screen Time, offer built-in, user-friendly reports of device and app activity, but rely on proprietary algorithms that define and categorize usage and can be updated without warning, limiting insight into what actually counts as screen time. Because Apple Screen Time algorithms are proprietary and Android Chronicle estimates were derived from reconstructed session events, it is difficult to determine whether observed differences in screen time estimates between data sources reflect true behavioral differences, sample differences, or platform-specific measurement differences. However, the purpose of this study was not to establish equivalence between Android and iOS estimates. Rather, we conducted separate analyses to provide reliability estimates for 2 distinct objective data sources commonly available to researchers studying smartphone use.

Passive sensing platforms such as Chronicle, Qustodio, and EARS can capture more granular event-level data and enable analyses beyond aggregate daily or hourly summaries [14,37]. However, these approaches often require complex processing pipelines and vary in validation, cost, and ease of implementation. A strength of the present study is the use of documented and reproducible processing procedures for both passive sensing and screenshot-based data. Future work should prioritize head-to-head comparisons of smartphone monitoring tools, validation of algorithmic definitions, and open standards for deriving digital behavior metrics.

### Limitations

Several limitations should be noted when interpreting these findings. First, the sample consisted of primary caregivers of young children, the majority of whom were female and already participating in observational studies about family screen time. This raises questions about selection bias, observer effects, and generalizability to other populations. Caregivers of preschool-aged children may also have more constrained and predictable daily routines than adolescents, older adults, or adults without young children [38]. In addition, because inclusion required 7 consecutive valid monitoring days, the analytic sample may overrepresent participants with greater adherence or more complete data capture. This concern may be particularly relevant for iOS Screenshot data, where included participants differed from excluded participants in race and education.

Second, Android Chronicle and iOS Screenshot data came from different platform and data source groups, and iOS Screenshot data were collected only in the cohort study. These groups differed in several demographic characteristics, including education, income, and employment. Because the study was designed to estimate reliability separately within each data source rather than to compare platforms directly, these differences were not adjusted for statistically. However, they limit interpretation of any descriptive Android-iOS differences in screen time estimates.

Third, a single 7-day monitoring window may not capture longer-term habitual smartphone behavior, as routines and occasional deviations may vary across weeks or seasons. For participants with more than 7 consecutive complete days available, the analytic window was defined as the first 7 days from the first qualifying consecutive sequence after cleaning; therefore, we cannot rule out the possibility that smartphone behavior differed during other portions of the monitoring period. This limitation in the monitoring window is important for interpreting the Spearman-Brown projections. The Spearman-Brown approach assumes that repeated monitoring days are exchangeable indicators of the same underlying behavior, but this assumption may be imperfect when behavior varies systematically by day type or over longer time frames. Recent work in physical activity measurement has shown that Spearman-Brown projections from short observation windows can overestimate longer-term aggregate reliability, and has proposed directly estimating reliability using repeated observation windows [39]. The present study was not designed to support this type of repeated-window analysis. Therefore, our findings should be interpreted as estimates of the number of valid days needed to estimate average daily smartphone duration within a 7-day monitoring period, rather than the number of days needed to characterize longer-term habitual smartphone use.

### Scope of Inference

The present findings apply specifically to total daily smartphone use duration measured over a 7-day monitoring window. Although total duration is a common and practical exposure metric, it does not capture other potentially important dimensions of smartphone behavior, including frequency of pickups, timing of use, fragmentation or clustering of use across the day, duration of individual sessions, app-specific use, or the content and context of interactions [40-42]. Reliability estimates for total daily duration should, therefore, not be assumed to generalize to these more granular behavioral metrics. Future work should examine how many monitoring days are needed to reliably estimate pattern-based and content-specific smartphone exposures, such as social media duration, messaging frequency, nighttime use, checking behavior, or fragmented vs sustained use.

### Conclusions

In conclusion, this study suggests that relatively few valid days of objective smartphone monitoring may be sufficient to estimate average daily smartphone screen time within a 7-day monitoring period among caregivers of preschool-aged children. These findings support the feasibility of brief monitoring protocols when some days are missing, invalid, or incomplete, helping to balance measurement reliability with participant burden and logistical constraints. However, these estimates should not be interpreted as establishing the number of days needed to measure longer-term habitual smartphone use or more granular smartphone behaviors beyond daily duration. Future studies with longer repeated monitoring windows should examine whether short-term reliability projections align with reliability across distinct weeks or longer time frames and across additional metrics.

## Supplementary material

10.2196/82846 Multimedia Appendix 1 Mean (SD) of daily smartphone screen time in minutes.

10.2196/82846 Checklist 1 STROBE checklist.

## Abbreviations

EARS: Effortless Assessment Research System
ICC: intraclass correlation coefficient
IRB: institutional review board

## References

[1] Mobile fact sheet. Pew Research Center.

[2] Ratan ZA, Parrish AM, Zaman SB, Alotaibi MS, Hosseinzadeh H (2021). Smartphone addiction and associated health outcomes in adult populations: a systematic review. Int J Environ Res Public Health.

[3] Wacks Y, Weinstein AM (2021). Excessive smartphone use is associated with health problems in adolescents and young adults. Front Psychiatry.

[4] Adamczewska-Chmiel K, Dudzic K, Chmiela T, Gorzkowska A (2022). Smartphones, the epidemic of the 21st century: a possible source of addictions and neuropsychiatric consequences. Int J Environ Res Public Health.

[5] Beynon A, Hendry D, Lund Rasmussen C (2024). Measurement method options to investigate digital screen technology use by children and adolescents: a narrative review. Children (Basel).

[6] Kaye LK, Orben A, Ellis DA, Hunter SC, Houghton S (2020). The conceptual and methodological mayhem of “screen time”. Int J Environ Res Public Health.

[7] Parry DA, Davidson BI, Sewall CJR, Fisher JT, Mieczkowski H, Quintana DS (2021). A systematic review and meta-analysis of discrepancies between logged and self-reported digital media use. Nat Hum Behav.

[8] Molaib KM, Sun X, Ram N, Reeves B, Robinson TN (2025). Agreement between self-reported and objectively measured smartphone use among adolescents and adults. Comput Hum Behav Rep.

[9] Júdice PB, Sousa-Sá E, Palmeira AL (2023). Discrepancies between self-reported and objectively measured smartphone screen time: before and during lockdown. J Prev (2022).

[10] Griffioen N, Scholten H, Lichtwarck-Aschoff A, van Rooij M, Granic I (2021). Everyone does it—differently: a window into emerging adults’ smartphone use. Humanit Soc Sci Commun.

[11] Parry D, Torth R (2025). Extracting meaningful measures of smartphone usage from android event log data: a methodological primer. Comput Commun Res.

[12] Barr R, Kirkorian H, Radesky J (2020). Beyond screen time: a synergistic approach to a more comprehensive assessment of family media exposure during early childhood. Front Psychol.

[13] Radesky JS, Weeks HM, Ball R (2020). Young children’s use of smartphones and tablets. Pediatrics.

[14] Lind MN, Kahn LE, Crowley R, Reed W, Wicks G, Allen NB (2023). Reintroducing the Effortless Assessment Research System (EARS). JMIR Ment Health.

[15] Reeves B, Ram N, Robinson TN (2021). Screenomics: a framework to capture and analyze personal life experiences and the ways that technology shapes them. Hum Comput Interact.

[16] Schnauber-Stockmann A, Scharkow M, Karnowski V, Naab TK, Schlütz DM, Pressmann P (2024). Distinguishing person-specific from situation-specific variation in media use: a meta-analysis. Communic Res.

[17] Bayer JB, LaRose R, Verplanken B (2018). The Psychology of Habit: Theory, Mechanisms.

[18] Wilcockson TDW, Ellis DA, Shaw H (2018). Determining typical smartphone usage: what data do we need?. Cyberpsychol Behav Soc Netw.

[19] Pan YC, Lin HH, Chiu YC, Lin SH, Lin YH (2019). Temporal stability of smartphone use data: determining fundamental time unit and independent cycle. JMIR mHealth uHealth.

[20] Koo TK, Li MY (2016). A guideline of selecting and reporting intraclass correlation coefficients for reliability research. J Chiropr Med.

[21] de Vet HCW, Mokkink LB, Mosmuller DG, Terwee CB (2017). Spearman-Brown prophecy formula and Cronbach’s alpha: different faces of reliability and opportunities for new applications. J Clin Epidemiol.

[22] Hart TL, Swartz AM, Cashin SE, Strath SJ (2011). How many days of monitoring predict physical activity and sedentary behaviour in older adults?. Int J Behav Nutr Phys Act.

[23] Aadland E, Ylvisåker E (2015). Reliability of objectively measured sedentary time and physical activity in adults. PLoS One.

[24] Aguilar-Farias N, Martino-Fuentealba P, Salom-Diaz N, Brown WJ (2019). How many days are enough for measuring weekly activity behaviours with the activPAL in adults?. J Sci Med Sport.

[25] Sasaki JE, Júnior JH, Meneguci J (2018). Number of days required for reliably estimating physical activity and sedentary behaviour from accelerometer data in older adults. J Sports Sci.

[26] da Silva SG, Evenson KR, Ekelund U (2019). How many days are needed to estimate wrist-worn accelerometry-assessed physical activity during the second trimester in pregnancy?. PLoS One.

[27] Parker H, Burkart S, Reesor-Oyer L (2022). Feasibility of measuring screen time, activity, and context among families with preschoolers: intensive longitudinal pilot study. JMIR Form Res.

[28] Reesor-Oyer L, Parker H, Burkart S (2022). Measuring microtemporal processes underlying preschoolers’ screen use and behavioral health: protocol for the Tots and Tech study. JMIR Res Protoc.

[29] Walch O, Alam U Arcascope/screen-scrape. GitHub.

[30] Chronicle-android-preprocessing. GitHub.

[31] Chronicle-preprocessed-cleaning. GitHub.

[32] Gamer M, Lemon J, Fellows I, Singh P (2005). irr: various coefficients of interrater reliability and agreement. CRAN: contributed packages.

[33] Spearman C (2010). The proof and measurement of association between two things. Int J Epidemiol.

[34] Baranowski T, de Moor C (2000). How many days was that? Intra-individual variability and physical activity assessment. Res Q Exerc Sport.

[35] Davison KK, Gicevic S, Aftosmes-Tobio A (2016). Fathers’ representation in observational studies on parenting and childhood obesity: a systematic review and content analysis. Am J Public Health.

[36] Domoff SE, Banga CA, Borgen AL (2021). Use of passive sensing to quantify adolescent mobile device usage: feasibility, acceptability, and preliminary validation of the eMoodie application. Human Behav Emerg Tech.

[37] Qustodio.

[38] Bianchi SM, Milkie MA (2010). Work and family research in the first decade of the 21st century. J Marriage Fam.

[39] Hilden P, Schwartz JE, Pascual C, Diaz KM, Goldsmith J (2023). How many days are needed? Measurement reliability of wearable device data to assess physical activity. PLoS One.

[40] Siebers T, Beyens I, Valkenburg PM (2023). The effects of fragmented and sticky smartphone use on distraction and task delay. Mob Media Commun.

[41] Toth R, Parry D, Emmer M (2025). From screen time to daily rhythms. J Quant Descr Digit Media.

[42] Yi H, Wu X, Liu X (2025). When smartphones fragment the mind: exploring the links between fragmented smartphone use and anxiety among college students through distraction and procrastination. Research Square.

[43] Smartphone-monitoring-reliability. GitHub.

