# Supplementary material for: Objective Smartphone Screen Time Monitoring Among Caregivers of Preschool-Aged Children: Reliability Study of Monitoring Duration
Source: JMIR Mhealth Uhealth. 2026 Sep 10;14:e82846. doi: 10.2196/82846 (PMC13561401; doi:10.2196/82846)
Supplement: Supplementary file 1 [file mhealth-v14-e82846-s002.docx]

STROBE Statement—checklist of items that should be included in reports of observational studies

|  | Item No. | Recommendation | Page  No. | Relevant text from manuscript |
| --- | --- | --- | --- | --- |
| **Title and abstract** | 1 | (*a*) Indicate the study’s design with a commonly used term in the title or the abstract | 2 | Abstract: “This secondary analysis included caregivers participating in two observational studies...” |
|  |  | (*b*) Provide in the abstract an informative and balanced summary of what was done and what was found | 2 | Structured abstract reports the background, objective, methods, sample, ICC results, monitoring-day recommendations, and conclusions. |
| Introduction | | | |  |
| Background/rationale | 2 | Explain the scientific background and rationale for the investigation being reported | 4–5 | Introduction explains limitations of self-report, advantages of objective measurement, within-person variability, and why ICC/Spearman–Brown methods are needed to determine monitoring duration. |
| Objectives | 3 | State specific objectives, including any prespecified hypotheses | 5 | “[We] estimate the minimum number of valid monitoring days required, within a 7-day monitoring period, to reliably estimate average daily smartphone screen time duration...” Separate estimates were produced for Android Chronicle and iOS Screenshot data. |
| Methods | | | |  |
| Study design | 4 | Present key elements of study design early in the paper | 5–6 | Methods identifies a secondary analysis of two observational studies: a pilot feasibility study and an ongoing longitudinal cohort study. |
| Setting | 5 | Describe the setting, locations, and relevant dates, including periods of recruitment, exposure, follow-up, and data collection | 5–6 | Non-random social-media recruitment across the Eastern United States; remote data collection. Pilot data: September 2020–February 2022 (30-day protocol). Cohort data: September 2024–May 2025 (14-day protocol). |
| Participants | 6 | (*a*) *Cohort study*—Give the eligibility criteria, and the sources and methods of selection of participants. Describe methods of follow-up  *Case-control study*—Give the eligibility criteria, and the sources and methods of case ascertainment and control selection. Give the rationale for the choice of cases and controls  *Cross-sectional study*—Give the eligibility criteria, and the sources and methods of selection of participants | 5–8 | Eligibility: primary caregiver of a child aged 3–5 years, smartphone owner, and able to read/speak English. Participants were selected from two parent studies; the analytic sample required at least seven consecutive complete objective-monitoring days after cleaning. |
|  |  | (*b*) *Cohort study*—For matched studies, give matching criteria and number of exposed and unexposed  *Case-control study*—For matched studies, give matching criteria and the number of controls per case | N/A | Not applicable; this was not a matched study. |
| Variables | 7 | Clearly define all outcomes, exposures, predictors, potential confounders, and effect modifiers. Give diagnostic criteria, if applicable | 6–10 | Primary outcome: day-level total smartphone screen time. Android: summed duration of device-unlock sessions per calendar day. iOS: daily Apple Screen Time total extracted from submitted screenshots. Data source and day type were fixed effects in the mixed model; participant was a random intercept. |
| Data sources/ measurement | 8* | For each variable of interest, give sources of data and details of methods of assessment (measurement). Describe comparability of assessment methods if there is more than one group | 6–9 | Android data came from Chronicle UsageEvents and were processed into sessions; iOS data came from Apple Screen Time screenshots with manual quality checks and scripted extraction. Pages 7–8 detail cleaning and validation; page 9 details ICC and Spearman–Brown assessment. |
| Bias | 9 | Describe any efforts to address potential sources of bias | 7–10, 15–16 | Prespecified cleaning excluded partial/incomplete days and truncated implausibly long events; screenshot validity checks were applied before aggregation. Included-versus-excluded demographics were compared. Limitations discuss selection bias, observer effects, adherence-related selection, and platform-group differences. |
| Study size | 10 | Explain how the study size was arrived at | 5, 8, 10 | No a priori sample-size calculation was reported. The study used available data from two parent studies and included participants meeting the prespecified requirement of seven consecutive complete days (229 of 316 screened). |

Continued on next page

| Quantitative variables | 11 | Explain how quantitative variables were handled in the analyses. If applicable, describe which groupings were chosen and why | 8–10 | Daily screen time was analyzed as a continuous measure (hours/minutes). Day type was weekday versus weekend. ICCs and projected monitoring days were calculated separately by data source; target reliability thresholds were 0.70, 0.80, and 0.90 and recommendations were rounded up to whole days. |
| --- | --- | --- | --- | --- |
| Statistical methods | 12 | (*a*) Describe all statistical methods, including those used to control for confounding | 8–10 | Linear mixed-effects model with data source, day type, and their interaction as fixed effects and participant random intercept. One-way random-effects absolute-agreement ICC(1,1), 500 participant-level bootstrap resamples, and Spearman–Brown projections were used. No confounder adjustment was performed because reliability was estimated within each data source. |
|  |  | (*b*) Describe any methods used to examine subgroups and interactions | 8, 10 | Analyses were conducted separately for Android and iOS. The mixed model tested a data-source-by-day-type interaction. Included-versus-excluded demographic comparisons were conducted within data source. |
|  |  | (*c*) Explain how missing data were addressed | 7–8 | Invalid or incomplete days were excluded using prespecified rules. Analyses required at least seven consecutive complete days; the first qualifying seven-day sequence was selected. No imputation was reported. |
|  |  | (*d*) *Cohort study*—If applicable, explain how loss to follow-up was addressed  *Case-control study*—If applicable, explain how matching of cases and controls was addressed  *Cross-sectional study*—If applicable, describe analytical methods taking account of sampling strategy | N/A | Not applicable; no loss-to-follow-up analysis, matching, or complex sampling strategy was used for this secondary reliability analysis. |
|  |  | (*e*) Describe any sensitivity analyses | 6, 12–13 | Sensitivity analysis used combined Android session and glance duration; the conservative three-day recommendation was unchanged (Table 4). |
| Results | | | | |
| Participants | 13* | (a) Report numbers of individuals at each stage of study—eg numbers potentially eligible, examined for eligibility, confirmed eligible, included in the study, completing follow-up, and analysed | 10 | 316 participants were available for screening; 229 were included. Android: 107 screened, 100 included, 7 excluded. iOS: 209 screened, 129 included, 80 excluded (Table 1). |
|  |  | (b) Give reasons for non-participation at each stage | 7–8, 10 | Exclusion reflected failure to obtain at least seven consecutive valid days after cleaning. Screenshot exclusions included wrong date, duplicates, unreadable images, or incomplete/unextractable hourly charts; Chronicle exclusions included partial days or prolonged inactivity gaps. |
|  |  | (c) Consider use of a flow diagram | 10 | No flow diagram was included; the relatively simple screening and inclusion counts are reported in Table 1. |
| Descriptive data | 14* | (a) Give characteristics of study participants (eg demographic, clinical, social) and information on exposures and potential confounders | 10–11 | Participant age, sex, race, Hispanic ethnicity, education, employment, and income are reported by data source in Table 2. |
|  |  | (b) Indicate number of participants with missing data for each variable of interest | 10–11 | Table 2 reports denominators for each characteristic (100 Android; 129 iOS); no missing values are shown for the reported characteristics in the analytic sample. |
|  |  | (c) *Cohort study*—Summarise follow-up time (eg, average and total amount) | N/A | Not applicable to the cross-sectional reliability analysis; the analytic period was a fixed seven consecutive days for every included participant. |
| Outcome data | 15* | *Cohort study*—Report numbers of outcome events or summary measures over time | N/A | Not applicable; the analysis did not examine incident outcome events over cohort follow-up. |
|  |  | *Case-control study—*Report numbers in each exposure category, or summary measures of exposure | N/A | Not applicable; this was not a case-control study. |
|  |  | *Cross-sectional study—*Report numbers of outcome events or summary measures | 10–13 | Summary measures include participant characteristics (Table 2), day-type/data-source estimates (page 12), and ICCs, variance components, and projected monitoring durations (Tables 3–4). |
| Main results | 16 | (*a*) Give unadjusted estimates and, if applicable, confounder-adjusted estimates and their precision (eg, 95% confidence interval). Make clear which confounders were adjusted for and why they were included | 12–13 | Mixed-model estimates are reported with SEs, 95% CIs, and P values. ICCs and projected monitoring days are reported with bootstrapped 95% CIs and variance components. No confounder-adjusted estimates were used. |
|  |  | (*b*) Report category boundaries when continuous variables were categorized | N/A | Not applicable; continuous participant variables or screen-time values were not categorized for the main analyses. |
|  |  | (*c*) If relevant, consider translating estimates of relative risk into absolute risk for a meaningful time period | N/A | Not applicable; relative-risk estimates were not calculated. |

Continued on next page

| Other analyses | 17 | Report other analyses done—eg analyses of subgroups and interactions, and sensitivity analyses | 10, 12–13 | Subgroup/data-source analyses, data-source-by-day-type interaction, included-versus-excluded comparisons, and the Android session-plus-glance sensitivity analysis are reported. |
| --- | --- | --- | --- | --- |
| Discussion | | | | |
| Key results | 18 | Summarise key results with reference to study objectives | 14, 16 | Discussion and conclusion summarize that two days met ICC ≥0.80 by point estimate and three by the conservative lower-bound criterion, with weekend representation recommended. |
| Limitations | 19 | Discuss limitations of the study, taking into account sources of potential bias or imprecision. Discuss both direction and magnitude of any potential bias | 15–16 | Limitations discuss selection and observer bias, adherence-related inclusion, demographic/platform differences, the single seven-day window, exchangeability assumptions, and restricted inference to total duration. Likely directions are described (e.g., overrepresentation of adherent participants and possible overestimation of longer-term reliability); exact magnitudes were generally not estimable. |
| Interpretation | 20 | Give a cautious overall interpretation of results considering objectives, limitations, multiplicity of analyses, results from similar studies, and other relevant evidence | 14–16 | Interpretation is explicitly limited to estimating average daily duration within a seven-day period, is compared with prior correlation and wearable-monitoring studies, and cautions against extrapolation to long-term habitual use or other smartphone metrics. |
| Generalisability | 21 | Discuss the generalisability (external validity) of the study results | 15–16 | Generalisability is discussed for predominantly female caregivers of preschool-aged children, with caution regarding adolescents, older adults, adults without young children, less-adherent participants, and more granular smartphone behaviors. |
| Other information | |  | | |
| Funding | 22 | Give the source of funding and the role of the funders for the present study and, if applicable, for the original study on which the present article is based | 17 | NIH support is reported (P20GM130420 and R01HD112311), with the statement that funders had no role in design, data collection, analysis, interpretation, manuscript preparation, or submission. |

*Give information separately for cases and controls in case-control studies and, if applicable, for exposed and unexposed groups in cohort and cross-sectional studies.

**Note:** An Explanation and Elaboration article discusses each checklist item and gives methodological background and published examples of transparent reporting. The STROBE checklist is best used in conjunction with this article (freely available on the Web sites of PLoS Medicine at http://www.plosmedicine.org/, Annals of Internal Medicine at http://www.annals.org/, and Epidemiology at http://www.epidem.com/). Information on the STROBE Initiative is available at www.strobe-statement.org.
